#  Effect of Medicinal Herbs on Primary Dysmenorrhoea- a Systematic Review 

**Published:** 2014

**Authors:** Parvaneh Mirabi, Seideh Hanieh Alamolhoda, Seddigheh Esmaeilzadeh, Faraz Mojab

**Affiliations:** a Fatemeh Zahra Infertility and Reproductive Health Research Center, Babol University of Medical Sciences, Babol, Iran.; b Midwifery and Reproductive Health Department, Shahid Beheshti University of Medical Sciences, Tehran, Iran.; c School of Pharmacy, Shahid Beheshti University of Medical Sciences, Tehran, Iran.

**Keywords:** Primary dysmenorrhea, Herbal medicine, JADAD Criteria, Randomized controlled trials

## Abstract

Conventional treatment for primary dysmenorrhoea has a failure rate of 20% to 25% and may be contraindicated or not tolerated by some women. Herbal medicine may be a suitable alternative. To determine the efficacy and safety of Iranian herbal medicine for primary dysmenorrhea when compared with placebo, no treatment, and other treatment.

Electronic searches of the Cochrane Menstrual Disorders and Dysmenorrhoea Group Register of controlled trials, Scopus, Google Scholar, Medline, Pubmed were performed to identify relevant randomized controlled trials (RCTs).

The study abstraction and quality assessment of all studies were undertaken following the detailed descriptions of these categories as described in the JADAD Criteria for Systematic Reviews of Interventions.

25 RCTs involving a total of women were included in the review. The review found promising evidence in the form of RCTs for the use of herbal medicine in the treatment of primary dysmenorrhoea compared with pharmacological treatment. However, the results were limited by methodological flaws. Further rigorous no penetrating placebo-controlled RCTs are warranted.

The review found promising evidence supporting the use of herbal medicine for primary dysmenorrhoea; however, results are limited by the poor methodological quality of the included trials.

## Introduction

There are about 8000 species of herbs in Iran, of which 1300 are endemic to Iran. Most consumers of medicinal plants are women who use them to alleviate problems such as menstrual disorders, mood disorders and menopause disorders, cyclical mastalgia and dysmenorrhea. They use these products more than chemical drugs because of being natural and having fewer side effects (1). 

The term dysmenorrhea refers to painful menstruation. Dysmenorrhea is a cramp labor-like pain in the lower abdomen that radiates to upper abdomen, waist and thighs and is sometimes accompanied by systemic symptoms like nausea, vomiting, diarrhea, headache and dizziness (2, 3). 

The prevalence of dysmenorrhea has been differently reported between 30 and 85%. Loudermilk expressed prevalence of dysmenorrhea between 50 and 80% with 10 to 18% of people having severe dysmenorrhea (4, 5).

In dealing with dysmenorrhea, medications such as prostaglandin synthesis inhibitors, nonsteroidal anti-inflammatory drugs and contraception pills are used irregularly because of fear of their side effects. Also, some of them have not been accepted in Iranian culture. Therefore, it seems necessary to find a new and simpler treatment for dysmenorrhea (6).

Some herbal products are effective on dysmenorrhea and its associated symptoms.Some plants are anti-spasmodic and some have a prostaglandin inhibitory effect. The mechanism of action of many herbal medicines is not completely understood.

Regarding the increasing demand for herbal medicine, many studies have been conducted on the analgesic effect of herbal extracts in Iran, but this is the first systematic review article in this field. This study aimed to systematically review and summarize analysis of clinical trials in this context and to investigate safety and efficacy of various methods for relieving dysmenorrhea.

## Experimental

*Methods*

All clinical trials of herbal products in treatment of primary dysmenorrheawere studied. Studies with inclusion criteria of women at reproductive age with moderate to severe primary dysmenorrhea and with regular menstrual periods as well as exclusion criteria of mild dysmenorrhea, irregular menstrual periods and obligation to use a particular drug entered this systematic review.

Databases such as Scopus, Google Scholar and Pubmed were searched and articles were evaluated according to Jadad Scale (7). This scale investigates articles based on probability of randomization error, patient’s follow–up and blinding. In this scale, the maximum score is 5. The papers which had scores of 3 or more were examined in this study. The results are presented qualitatively.

## Results

The systematic search primarily investigated clinical trials of herbal products (investigating abstracts) in Iran. Then, according to Jadad Scale, 25 studies were investigated in the secondary study (investigating the whole article).These clinical trials included the following: 

*Foeniculum vulgare *(8 articles) (Table 1), *Mentha piperita *extract (1 article), *Zataria multiflora *(1 article), *Valeriana officinalis *(2 articles), *Cinnamomum zeylanicum *(1 article), *Zingiber officinale *(2 articles), *Matricaria chamomoilla *(1 article), *Stachys lavandulifolia *(2 articles), *Echinophora platyloba *(1 article), *Cuminum cyminum *(1 article), *Vitex agnus-castus *(1 article), Menstrogol^®^ (2 articles), Menastil^®^ (1 article), *Achillea willhemsii *(1 article) (Table 2).

In terms of blinding, there were 4 triple-blind studies, 8 randomized double-blind clinical trials, 5 randomized single-blind clinical trials and 7 unblinded studies.

**Table 1:** Clinical trials have been conducted on the impact of *Foeniculum vulgar*e on primary dysmenorrheal (PD).

Jadad score	Side effects and possible reaction	Results	Measure	Participants	Control group	Experimental group	method	Authors (year)	Ref
Cannot be calculated	Not mentioned	The pain Reduced by *F. vulgar*e and *M. chamomilla.*	VAS	60 students		1^st^ cycle as control, cycles 2 and 3 *Foeniculum vulgar*, cycles 4 and 5 *Matricaria Chamomilla*	A clinical trial (before - after)	Yazdani (2004)	16
Cannot be calculated	Increased bleeding in 1 case	The pain reduced by *F. vulgare *and Mefenamic acid.	VAS	30 students 15-24 years old with PD (moderate and severe)		1^st^ cycle without drug, 2^nd ^cycle Mefenamic Acid 250mg/QID, 3^rd ^cycle 25 drops *F. vulgar *from 1^st ^da**y.**	A clinical trial witnessed 3 cycles	Namvar-Jahromi (2003)	13
5	Three groups did not differ	more reduction in pain intensity with *F. vulgare *2%	VRS	60 single female students 17-25 years old	placebo	*F. vulgar*e oil 1% or 2% and placebo cross over during 3 cycles	A double-blind, randomized clinical trial, 3 cycle**s**	Khorshidi (2002)	11
5	Uncomplicated	Reduction pain in *F. vulgar*e group compared to the placebo group. Systemic symptoms in the two groups did not differ	VAS	130 single female students 17-25 years old who experienced moderate to severe dysmenorrhea	placebo	*F. vulgar*e capsules daily for 5 days during the first 3 day**s**	A blind, randomized clinical trial, 2 cycle**s**	Tork-zahrani (2007)	10
4	Uncomplicated	The pain reduced by *F. vulgar*e and Mefenamic acid than placebo group. There was no difference between the 2 drugs, reduction systemic symptoms were seen in *F. vulgare *group.	Questionnaire and VAS	120 single students 17-25 years old who experienced moderate -severe dysmenorrhea	Mefenamic acid 250 mg/QID and placebo (drop)	*F. vulgare *20 to 30 drops every 4 to 8 hours with onset menstruation	A blind, randomized clinical trial, 2 cycle**s**	Nazarpoor (2007)	15
4	Uncomplicated	not difference between the 2 groups	VMSS	110 female students 13 years and older		1^st^ group 30 drops *F. vulgar*e QID with onset pain during 3 days, 2^nd ^group Mefenamic Acid 250 mg/QID with onset pain during 3 days.	A blind, randomized clinical trial, 1 cycle before -2 cycles afte**r**	Modarres-nejad (2006)	14
5	Uncomplicated	The pain reduced by *F. vulgar*e group than placebo	VAS	60 single students 18-25 years old who experienced moderate–severe dysmenorrhea	placebo	30 drops *F. vulgare *extract TID for 3 days before menstruation and in the first 3 days of menstruation.	double-blind, randomized, placebo controlled trial	Delaram (2001)	12
5	Uncomplicated	Mean pain scores were similar between the 2 groups before the inter- vention, pain intensity decreased in 2 groups, but this reduction in *F. vulgare *was more than *Echinophora platyloba*	VAS	60 single students 18-25 years old who experienced moderate–severe dysmenorrhea		A group, 30 drop *Echinophora platyloba *extract TID for 3 days before menstruation and in the first 3 days of menstruation, Another group *F. vulgar*e extract in same condition	A clinica**l **trial before – after, 2 cycles	Delaram (2011)	57

**Table 2:** Clinical trials have been conducted on the impact of other herbs on primary dysmenorrheal (PD).

**Jdad score**		**Side effects and possible reaction**		**results**		**Measure**	**Participants**			**Control group**	**Experimental group**	**method**			**Authors (year)**	**Ref.**		
5		Uncomplicated		Herbal extract was more effective than mefenamic acid in pain reduction		VAS	180 students 18-27 years old			placebo	One group 500 mg/TID Menstrugol^®^ (saffron, celery and aniseed) for first 3 days of menstruation, another group Mefenamic acid 250 mg/TID	double-blind, randomized, placebo controlled trial 3 cycles			Khodakarami (2009)	23		
4		Uncomplicated		There was no difference between the two groups.		VAS	161 students 17-30 years old			Placebo	Menstrugol^® ^(saffron, celery and aniseed)/TID for first 3 days of menstruation, another group Mefenamic acid 250 mg/ TID	double-blind, randomized, placebo controlled trial, 3 cycles			Khodayari (2004)	24		
4		Two patients were excluded due to nausea.		70**% **reduction in pain intensity in experimental group versus 6% in control.		VAS	60 students with moderate or severe dysmenorrhea			placebo 40 drops daily for 3 months from the 1^st^ day of menstruation	Vitagnus^®^ (*Vitex agnus-castus*) 40 drops daily for 3 months from the 1^st ^day of menstruation	double-blind, randomized, placebo controlled trial, 3 cycles			Shahhosseini (2006)	53		
5		Uncomplicated		There was no difference between the 2 groups.		VMSS	150 students			One group Mefenamic acid 250 mg/QID, another group Ibuprofen 400 mg/QID	250 mg ginger/QID for 3 days	double-blind, randomized, placebo controlled trial, 3 cycles			Ozgoli (2007)	18		
4		Uncomplicated		Reduction the severity of dysmenorrhea and signs and pain killer and menstrual bleeding		VAS	72 single female students 18-26 years old with moderate and sever PD			Baby oil	A topical lotion Menastil daily maximum of 3 doses in the first 2 days of menstrual cycles within 2	double-blind, randomized, placebo controlled trial, 2 cycles			Kariman (2007)	21		
5		Uncomplicated		Reduction the severity of dysmenorrhea in valerian group than placebo. The total scores of the systemic manifestations decreased after the intervention, but there was no significant difference between the groups, with the exception for syncope.		VAS and VMSS	100 students 18-24 years			Placebo (starch) 3 days beginning at the onset of menstruation	250 mg valerian root/TID for 3 days beginning at the onset of menstruation, for 2 consecutive menstrual cycles	randomized clinical trial cycles 2			Mirabi (2011)	32		
4		Not mentioned		*Zataria multiflora *1% or 2%, compared with placebo in reducing dysmenorrhea		VAS and VMSS	108 students 18-25 years old with dysmenorrhea (moderate or severe)			Placebo	One group 25 drop *Zataria multiflora *1%, another group 25 drop *Z. multiflora *2% onset dysmenorea	double-blind, randomized, placebo controlled trial, 3 cycles			Irvani (2009)	46		
3		Uncomplicated		There was no difference between the 2 groups.		Questionnaire, VMSS and Criterion Andrish Mylsvm	100 single female students 18-22 years old who experienced moderate–severe dysmenorrhea.			**-**	1^st^ cycle without drug, for 2^nd ^cycle 40 drops *Mentha piperita *every hour in case, another group 400 mg Ibuprofen every 4 hours in first 3 days of menstruation	Randomized clinical trial, 2 cycles			Amoyi-rokn abaad (2012)	51		
5		Uncomplicated	Reduction in pain compared to placebo group, cinnamon Higher. Systemic symptoms were not different between the two groups		Questionnaire and VMSS		47 single female students 18-30 years old who experienced moderate–severe dysmenorrhea.	Placebo			5 capsules 420 mg cinnamon for first 3 days of menstruation in 2 consecutive cycles	double-blind, randomized, placebo controlled trial, 2 cycles			Akhavan-amjadi (2009)		37
Cannot be calculated	Uncomplicated		During the time of dysmenorea and pain Intensity after consumption *Stachys lvandulifolia *significant difference was observed. Systemic symptoms, the scores did not differ from factors other than changes in the nervous.		VAS and VMSS		50 single female students 18-25 years old				*Stachys lvandulifolia *(5 g infusion)/TID in starting bleeding	Clinical trial, 2 cycles			Mirabi (2012)		42
4	Uncomplicated		There was no difference between the 2 groups.		Questionnaire and VAS		102 students				1^st^ group valerian 250 mg within 2 to 3 days of menstruation cycles, and the 2^nd ^group 250 mg Mefenamic acid	Randomized clinical trial			Jenabi (2009)		27
5		Uncomplicated	The duration and severity of primary dysmenorrhea in ginger. To be significantly reduced compared with placebo.		VAS and VMSS		Mean age 21 years, 78 female students with moderate to severe dysmenorrhea	Placebo			500 mg ginger TID in first 3 days of menstruation	triple-blind, randomized, placebo controlled trial			Rahnema (2011)		19
5		Two patients were excluded due to nausea	Pain severity in *Echinophora platyloba, *to be significantly reduced compared with placebo**.**		VAS		60 students	Placebo			*Echinophora platyloba *extract 30 drops TID for 3 days before the start of menstruation and in the first 3 day of menstruation	A clinical trial before-after 2 cycles			Delaram (2010)		56
5	Uncomplicated		In both Mefenamic acid and *Matricaria chamomilla *(MC), menstrual pain decreased after 2 cycles of treatment, but this reduction was seen in MC.		Questionnaire and VAS		80 single female students 20-30 years old who experienced moderate–severe dysmenorrhea				*Matricaria chamomilla *(400 mg) within 1^st ^day or a day before the start of menstrual bleeding for 2 consecutive cycles and for 3 days 800 mg QID in each, and the 2^nd ^group with the same group of 500 mg Mefenamic acid in the same way before	triple-blind randomized cross over clinical trial			Modaress (2011)		28
Cannot be calculated	Uncomplicated		Among consumer groups *Cuminum cyminum*, 2 doses TID had the lowest pain. Also reduction in pain intensity between the 1^st ^and 2^nd ^cycles of treatment. Significantly differences between the intervention groups than previously reported.		VMSS		100 students 14-18 years old				5 groups: three groups consumed *C. cyminum *(65 mg/TID, 130 mg/TID and 65 mg per 12 h), one group consumed placebo and the last group took Mefenamic acid		Randomized clinical trial 3 cycles		Tavasoli (2002)		22
Cannot be calculated	Not mentioned		Pain during the first 3 days of treatment cycles compared with cycles without treatment significantly reduced.		Questionnaire and VAS		70 students with moderate dysmenorrhea				1 capsule of *Achillea willhemsii *extract TID	Randomized clinical trial			Satar-zadeh (2009)		59
Cannot be calculated	Uncomplicated		After consumption *Stachys lvandulifolia *significant difference was observed. The total scores of the systemic manifestations decreased. Decreased after the intervention, but there was no significant difference between the groups, with the exception for nervous.		VAS and VMSS		50 single female students 18-25 years old				10 g of infused plant powder TID (2 days before and 3 days after the onset of pain)			Clinical trial, 3 cycles	Olfati (2011)		41

*Foeniculum vulgare*

*F. vulgare *is from Apiaceae family whose root, leaf and fruit can be used. This plant has been used in Iranian traditional medicine for many centuries and has anti-inflammatory, analgesic and antispasmodic effects (8).

The mechanism of its analgesic effect is explained in two ways:

1. The essential oil of this product has analgesic effects in uterus by inhibiting contractions induced by oxytocin and prostaglandins.

2. This product facilitates discharge of blood in shorter time, which reduces dysmenorrhea (9).

Eight studies have examined the effect of *F. vulgare *on primary dysmenorrheal. Three of them compared the effect of *F. vulgare *with placebo (10-12), two compared the effect of *F. vulgare *with mefenamic acid (13 and 14) and one compared the effect of *F. vulgare *and placebo with mefenamic acid (15).

In one study, the effect of *F. vulgare *and *M****. ****chamomoilla *(16) and in another, the effect of *F. vulgare *and *E. platyloba *were compared (17). Khorshidi *et al. *showed that *F. vulgare *essential oil was useful in reducing pain and systemic symptoms of primary dysmenorrheal compared with placebo (11), but in the study by Zahrani *et al., F. vulgare *did not affect systemic symptoms (10). Jahromi *et al*. compared *F. vulgare *and mefenamic acid for treating primary dysmenorrhea, and concluded that both drugs were significantly effective in reducing dysmenorrhea in comparison with the control group (13).

*F. vulgare *has been effective in reducing severity of dysmenorrhea in all studies; in comparative studies with mefenamic acid, there has been no significant difference between the two groups in terms of reducing dysmenorrheal (Table 1).

*Zingiber officinale*

*Z. officinale *has a long history in medicine and is one of the potent inhibitors of prostaglandins (via cyclooxygenase inhibition). In some sources, one of the traditional uses of *Z. officinale *is for treating dysmenorrhea. (18)

In a double-blind clinical trial, the effect of *Z. officinale *was compared with that of mefenamic acid and ibuprofen for treatment of primary dysmenorrhea and there was no significant difference in the improvement of primary dysmenorrhea in three groups of *Z. officinale *(64%), ibuprofen (66%) and mefenamic acid (58%). In other words, *Z. officinale *was effective in the treatment of primary dysmenorrhea like two conventional chemical drugs (18). In another study, rhizome powder of *Z. officinale *was effective for reducing pain intensity in comparison with the placebo (19).

Menastil^®^

Menastil^®^ containing calendula oil and mint essential oil. Menastil^®^ prevents transmission of messages from nerve cells of uterus to brain by shortening axons of nerve cells and leads to late transfer of pain messages from brain to uterus (20).

In a clinical trial, topical use of Menastil lotions was more effective than placebo in reducing severity of dysmenorrhea and its symptoms. Furthermore, sedative consumption and menstrual bleeding decreased (21).

*Cumminum cyminum*

*C. cyminum *is useful for treatment of gastrointestinal diseases, delayed and painful menstruation. In a randomized clinical trial, patients were divided to 5 groups. Three groups consumed 65 mg of *C. cyminum *capsules (1 capsule/TID, 2 capsules/TID and, the 3^rd ^group, 1 capsule/BID), one group consumed a placebo capsule and the last group took mefenamic acid. Finally, among consumers of *C. cyminum*, those who had 2 capsules/TID had minimal pain, which was equal to those who consumed mefenamic capsule per 8 h. Furthermore, there was a significant differenceamong the three groups in terms of reducing pain intensity in the first and second periods after the treatment compared withthat before intervention (22).

Menstrugol^®^ (saffron, celery and aniseed)

In a clinical trial, an herbal capsule (Menstrugol^®^) containing extract of 3 plants (saffron, celery and aniseed) worked for primary dysmenorrhea and had better and more proper effects than mefenamic acid (23). 

In another study, this extract was more effective than placebo on severity of dysmenorrhea (24).

*Matricaria chamomilla*

*M. chamomoilla *is a traditional herbal drug whose extract has anti-inflammatory and antispasmodic effects. It also has a sedative and anti-anxiety effect (25). Many studies have shown the rate of improvement in dysmenorrhea is more for groups using *M. chamomoilla *than placebo. *M. chamomoilla *is more effective on relieving dysmenorrhea if it is used before pain begins (26)

Three studies examined the effect of *M. chamomoilla *on primary dysmenorrhea. In the first study, after prescribing *M. chamomoilla *tea, the rate of anxiety inthe interventiongroup was significantly different from those in the control group after one month (27). In the second study, mefenamic acid was compared with *M. chamomoilla *in across over treatment which reported that *M. chamomoilla *was more effective than mefenamic acid in reducing pain (28).

Another study compared the effects of *M. chamomoilla *with *F. vulgare*. Consumption of *M. chamomoilla *and *F. vulgare *had a significant effect on three symptoms of premenstrual syndrome and dysmenorrhea in which *M. chamomoilla *was more effective on pelvic and abdominal pain, depression and anger and *F. vulgare *was more effective on reducing fatigue and lethargy (29).

*Valeriana officinalis*

*V. officinalis *has been traditionally used as a menstruating and sedative drug since 11^th^ century (30). Its root and rhizome have valerian essential oil which contains valepotriats. Root of *V. officinalis *is used as a diuretic, sedative and muscular antispasmodic and valerenic acid of its root has antispasmodic properties (31). Also, the effect of anti-spasmodic valterate, isovalterate on ileum smooth muscle has been confirmed (32). *V. officinalis *inhibits contractions of cell depolarization well and blocks calcium channels (33).

Two studies have been conducted on *V. officinalis*, one of which compared the effect of its root with placebo (34) and another compared the effects of *V. officinalis *with mefenamic acid (35). In the first study, *V. officinalis *was effective on reducing pain compared to placebo, and in the second study, it had a similar effect to that of mefenamic acid. In one research, systemic symptoms of dysmenorrhea reduced after taking *V. officinalis *capsules compared to pre-intervention, but the difference was the same as placebo group, except for severity of fainting variable which was significantly different between *V. officinalis *and placebo groups (32).

In traditional medicine, *V. officinalis *is known as a menstruating herb, but in a clinical trial, *V. officinalis *had no effect on duration and severity of bleeding (6).

*Cinnamomum zeylanicum*

*C. zeylanicum *is from Laurceae family. *C. zeylanicum *contains mucilage, tannin, a pigment, calcium oxalate, sugar, essential oil and resin. Its physiological effect is attributed to its essential oil and tannin. The main component of cinnamon essential oil is cinnamaldehyde and the essential oil from its bark contains 55 to 57% of cinnamaldehyde and 5 to 18% of eugenol. It has been reported that *C. zeylanicum *has an antispasmodic effect. Eugenol can also inhibit biosynthesis of prostaglandins and affect inflammation (36).

In a triple-blind study, *C. zeylanicum *capsule was compared with placebo. In this study, the effect of *C. zeylanicum *on severity of dysmenorrhea was more than that of placebo. The difference was statistically significant (37).

*Stachys lavandulifolia*

*S. lavandulifolia *grows in the mountains of Iran and can be abundantly found in Alvand Mountain. This plant is effective in spasm treatment, is a menstruating agent in women, increasessexuality andcauses abortion and is a pain killer (38).

According to previous studies, *S. lavandulifolia *can inhibit production of prostaglandins (mediators of pain). *S. lavandulifolia *extract is also used for stomach pain and painful menstruation (38-40). In a study, 10 g of infusedplant powder was prescribed three times (in Iraniantraditional medicine) and no significant difference was observed in duration of pain and severity of pain before and after using *S. lavandulifolia *(41).

In another study, *S. lavandulifolia *affected duration and severity of menstrual pain, but it did not affect systemic symptoms; only reduced diarrhea (42).

*Zataria multiflora*

*Z. multiflora *is from mint family and its essential oil is thymol and carvacrol. Investigation on ancient physicians’ studies has shown that *Z. multiflora *was used to treat seizures, respiratory diseases, smooth muscular spasm and bloating (43). The most common effect of *Z. multiflora *is its antispasmodic effect on smooth muscles and its antimicrobial property (44).

Van den Broeke stated that flavonoid of *Z. multiflora *can inhibit contractions induced by cell depolarization and blocks calcium channel (45).

In a study, the participants were randomly divided to three groups. The first group was treated by placebo, the second by 1% of *Z. multiflora *essential oil and the third by 2% of *Z. multiflora *essential oil. The maximum pain reduction was reported in the group with 2% of essential oil (46).

In another study, *Z. multiflora *leaves had a similar effect to that of mefenamic acid on pain reduction (47).

*Mentha piperita*

*M. piperita *is used in traditional medicine. The essential oil of this herb is colorless or pale yellow or greenish yellow with odor and pungent taste, obtained from distillation of flowers and fresh twigs.

Experimental studieshave shown that *M. piperita *oil inhibits contractions induced by cell depolarization and blocks calcium channels and has antispasmodic properties for smooth muscles (48-50).

In a study, the effect of Supermint^®^ (mint extract) was compared with that of ibuprofen, both of which reduced pain (51).

*Vitex agnus-castus*

One of the herbal medicines used for treating menstrual disorders is an herbal drop called Vitagnous^®^ and a combination which is derived from *V. agnus-cactus *plants. This plant has a dopaminergic effect. Important combinations of *V. agnus-castus*, especially its essential oil, affect hypothalamus-pituitary axis and decrease secretion of FSH, release of LH and increase progesterone. Indeed, physiological and pharmacological effects of this drug cause human body to naturally balance hormonal reduction or increase (52)

In one study, the effects of Vitagnous^®^ were compared with those of placebo and it was reported that Vitagnous^®^ was more effective than placebo in terms of reducing pain intensity (53).

*Echinophora platyloba*

*E. platyloba *is from Apiaceae family and is one of the endemic plants in Iran.The growth of this plant is mostly distributed in Mediterranean region. Results have shown that the extract of this plant can reduce muscle contraction. Antispasmodic effects of this plant can completely inhibit intestinal irritability (54-55).

Two studies have been conducted in this field, one of which compared the effects of *E. platyloba *extract with those of placebo (56) and another compared the effects of *E. platyloba *and fennel on decreasing severity of dysmenorrheal (57). In the first study, *E. platyloba *extract had a significant difference with placebo in terms of reducing pain intensity, and in the second study, extracts of *E. platyloba *and *F. vulgare *could reduce severity of dysmenorrhea during the treatment. The effect of *F. vulgare *in this case was more than that of *E. platyloba*.

*Achillea willhemsii*

*A. willhemsii *is a plant with anti-prostaglandin effect. It is from Asteraceae (Compositae) family and its anti-inflammatory property of flavonoids in *A. willhemsii *is due to its inhibitory effect on metabolism of arachidonic acid (58).

In an experimental study, a capsule containing extract of *A. willhemsii *was prescribed to 70 college students with dysmenorrhea every 6 h. They could reduce the frequency of consumption or dose of drug by 50%, but total daily consumption of capsule should not exceed 4. The results showed that duration of pain in the first three days with treatment reduced significantly compared with the cycles without treatment (59).

## Discussion

The aim of this study was to evaluate the efficacy of different herbal remedies on the intensity of primary dysmenorrhea. Most acceptable studies have examined herbal and nutritional treatments. Among them, the number of studies on the use of *F. vulgare *was more than others. Nineteen trials took 3 to 5 scores of Jadad. In five studies, Jadad score could not be calculated due to the trial type.

In these studies, randomizationmethods, blinding, follow-up, concealment allocation and intention of treatmenthave been used according to Jadad Scale. However, there is not a detailed description of randomization, blinding andfollow-up in most studies. Furthermore, there is the possibility of biased publication because of positive results of all the studiesand there is not even one publication with negative results.

Different types of *F. vulgare *including essence, extract and capsule have been compared with placebo or sedative drugs such as mefenamic acid or other herbs (*M. chamomilla *and *E. platyloba*) in order to control pain and in none of these studies, side effects of using the plant have been reported. So, considering numerous studies on *F. vulgare*, it is suggested as a safe and efficacious plant. It seems that it can be used in the treatment of dysmenorrhea. In a study by Jahromi *et al*., one patient had increased bleeding (13), but a study by Akhavan et al. showed that *F. vulgare *did not increase intensity of bleeding and duration of menstruation (60).

There is only one study on the plants such as *C. cyminum, C. zeylanicum, *Menastil, *E. platyloba*, *V. agnus-castus, Z. multiflora*, *M. piperita *and *A. willhemsii*. Although the results of these studies are positive and these plants are effective on reducing dysmenorrhea, there are not enough studies in this area. Considering their safety, more powerful studies are needed to examine their side effects. It is also suggested to compare different routes of administration for these plants to determine the best route.

Although herbs such as *Z. officinale, S. lvandulifolia M. chamomilla *and *V. officinalis *have proved effective, further clinical trials are necessary with the same scale for measuring pain, investigating possible side effects, observing blinding rules and randomization so as to provide a definitive conclusion about their effective use and dose.
